# Low Power Contactless Voltage Sensor for Low Voltage Power Systems

**DOI:** 10.3390/s19163513

**Published:** 2019-08-11

**Authors:** Antonio Delle Femine, Daniele Gallo, Carmine Landi, Alessandro Lo Schiavo, Mario Luiso

**Affiliations:** Department of Engineering, University of Campania “Luigi Vanvitelli”, 81031 Aversa (CE), Italy

**Keywords:** power system measurements, voltage measurement, contactless, low power, sensor, non-sinusoidal conditions

## Abstract

Contactless measurements represent the desirable solution in many contexts, where minimal cabling is required or, in general, cabling is not possible. This paper presents a new contactless voltage sensor for low voltage power systems. It is based on a contactless capacitive probe, which surrounds the power cable. It has two concentric electrodes insulated by a shield. A low power analog conditioning circuit evaluates the power line voltage by measuring the current in one of the capacitances of the probe. All the single stages of the circuit have been designed by using low-power rail-to-rail operational amplifiers, supplied at 3.3 V, in order to minimize the power absorption. The sensor has been characterized in various conditions, with sine waves and distorted signals, varying the frequency and the harmonic distortion. The influence of the current, flowing into the power cable, on the voltage measurement has been evaluated too. It shows a good accuracy (lower than 0.3%) from 100 V to 300 V, with a power consumption less than 5 mW.

## 1. Introduction

The demand of smart measurement systems and innovative sensors based on Internet of Things (IoT) methods has increased thanks to advances in the fields of low power electronics, information technology, and communications [1]. It is possible to realize new devices for different applications with a large variety of sensing elements able to transmit the information with minimal maintenance and without the adoption of wires [2].

However, in order to allow the widespread diffusion of IoT devices, they must be simple to use and to install. In this context, measuring devices adopting contactless sensing principles represent enabling technologies for the IoT.

Contactless voltage and current sensors have numerous applications in electrical power systems. They are used for state monitoring of high voltage transmission line [2] appliances, load monitoring applications for smart home and industries [3,4,5,6,7,8], smart metering applications [9] and for condition monitoring and recording of electrical rotating machines [10]. Established methods are available for non-invasive current measurement for direct current (DC) and alternating current (AC) applications such as Hall effect current sensors [11] and current transformers [12,13,14,15] with a high degree of accuracy. There are few non-invasive AC measurement schemes using capacitive coupling-based measurement of transmission line voltage [16,17,18,19]. Even though some of the cited solutions present high accuracy and some have been employed for IoT applications, they all are characterized by high power consumption (related to IoT applications), which makes them unprofitably usable for the IoT paradigm. In fact, the high power demand makes necessary the use of a robust power supply or, alternatively, a battery with a high capacity, increasing the dimensions and cost—both of these characteristics are against the widespread diffusion of the solutions.

Starting from these considerations, in this paper, a new power line contactless voltage sensor (hereafter CVS) is presented. It has a very low power consumption, compared to most solutions presented in literature, allowing the use of an energy harvester for the power supply [20]. Moreover, it has small dimensions and it is lightweight. All of these features make it very attractive for the IoT applications. A similar approach is presented in Reference [19]; however, it makes use of non-linear devices and phase shifters, which have power consumption not suitable for IoT applications and, moreover, compromise the accuracy of the whole device.

The measurement principle and a preliminary characterization of a prototype was already presented in Reference [21]. Here, the circuit design is discussed and a thorough experimental characterization, in the most common situations of a low voltage power system, is presented. Moreover, thanks to a specifically realized measurement setup, the influence of a current, flowing into the power cable, on the performance of the CVS is also evaluated.

The paper is organized as follows. Section 2 discusses the measuring principle and the architecture of the proposed system. Section 3 presents the circuit design, whereas the realization of the prototype is shown in Section 4. Section 5 deals with the experimental characterization of the prototype: the measurement setup, the performed tests and the experimental results are described. Finally, Section 6 draws the conclusions.

## 2. The Proposed System

**Figure 1:**
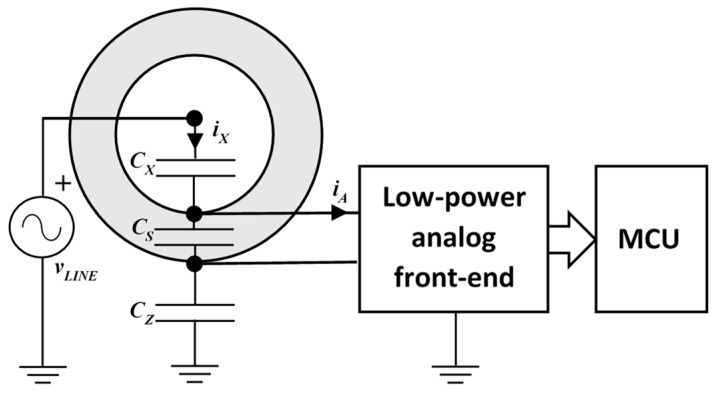
Scheme of the low-power contactless voltage measurement system.

The contactless system for power-line voltage measurement is made up of a capacitive probe, a low-power analog front-end and a low-power microcontroller, as shown in Figure 1.

The contactless capacitive probe surrounds the line cable with two metallic surfaces, the inner electrode and the outer shield. This arrangement creates a capacitance $C_{X}$ between the line and the electrode, a capacitance $C_{S}$ between the electrode and the shield, and a capacitance between the shield and the earth.

As the shield is driven by the low-power analog front-end at the same voltage of the electrode, no current flows through the capacitance $C_{S}$. Thus, the current, $i_{X}$, flowing through the capacitance $C_{X}$, is equal to the current, $i_{A}$, which is detected by the analog front-end in order to determine the line voltage. Unfortunately, the current $i_{X}$ depends not only on the line voltage, $v_{LINE}$, but it also depends on the value of the capacitance of $C_{X}$ itself. In order to estimate the value of $C_{X}$, the analog front-end generates a reference signal at frequency $\omega_{R}$, different from the line frequency $\omega_{L}$, and injects it into the electrode. In this way, the detected current, $i_{A}$, includes both a line frequency component and a reference frequency component, which depends only on the unknown value $C_{X}$. By separating the two frequency components and by estimating the value of the capacitance $C_{X}$, the measurement system is able to estimate the amplitude of the fundamental component of the line voltage, $v_{LINE}$.

**Figure 2:**
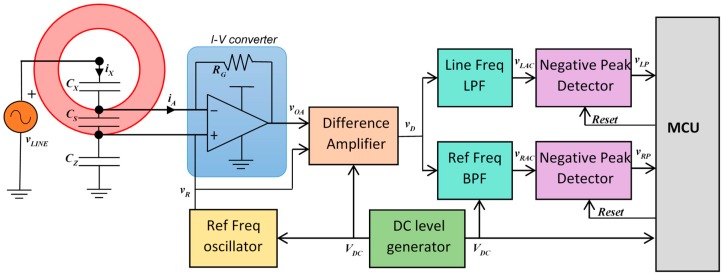
Block diagram of the low-power analog front end.

The principle of operation of the system in Figure 1 led to interesting implementations presented in literature [19,22]. Differently from those implementations, here the analog front-end is designed in order to minimize the power consumption, aiming at its use in a wireless sensor node, possibly supplied by an energy harvesting source [20]. In order to reach the target, a very simple analog processing circuit, whose block diagram is shown in Figure 2, is here proposed.

The oscillator generates the sinusoidal reference signal, $v_{R}$, having amplitude $V_{R}$, frequency $\omega_{R}$, and a DC component $V_{DC}=V_{DD}/2$, which is created by the DC level generator. The sinusoidal reference signal drives the non-inverting op-amp terminal, connected to the probe shield, and by virtue of the virtual short-circuit principle, also the inverting op-amp terminal is driven at the same voltage $v_{R}$. Thus, the current $i_{X}=i_{A}$, flowing through the capacitance $C_{X}$, depends on both the line voltage, $v_{LINE}$, and on the reference voltage, $v_{R}$.

By applying the superposition principle to the voltage sources $v_{LINE}$ and $v_{R}$, it is possible to calculate the output voltage of the operational amplifier of the input stage, that is

(1) $$v_{OA}=-R_{G}\;i_{A}=-s\;R_{G}C_{x}\;v_{LINE}+s\;R_{G}C_{x}\;v_{R}+s\;R_{G}C_{in}\;v_{R}+v_{R}$$

where $R_{G}$ is the gain resistance of the input stage and $C_{in}$ is the input capacitance at the inverting terminal of the operational amplifier. The difference amplifier performs the difference between the voltage in (1) and the reference voltage. It also adds the *DC* voltage $V_{DC}$, in order to ensure a proper bias of the output voltage. Thus, the output of the difference amplifier can be written as

(2) $$v_{D}=-s\;R_{G}C_{x}G_{D}\;v_{LINE}+s\;R_{G}C_{x}G_{D}\;v_{R}+s\;R_{G}C_{in}G_{D}\;v_{R}+V_{DC}$$

where $G_{D}$ is the gain of the differential amplifier.

The line-frequency low-pass filter (LPF) and the reference frequency band-pass filter (BPF) separate and amplify the two frequency components, generating the signals $v_{LAC}$ and $v_{RAC}$, respectively, i.e.,

(3) $$v_{LAC}=V_{DC}-s\;R_{G}C_{x}G_{L}\;v_{LINE}$$

(4) $$v_{RAC}=V_{DC}+s\;R_{G}C_{x}G_{R}\;v_{R}+s\;R_{G}C_{in}G_{R}\;v_{R}$$

where $G_{L}$ and $G_{R}$ are the overall gains for the line signal and for the reference signal, respectively.

Finally, the negative peak detectors provide, as output, the signal negative peaks, $V_{LP}$ and $V_{RP}$, of the signals $v_{LAC}$ and $v_{RAC}$, respectively

(5) $$V_{LP}=V_{DC}-\omega_{L}\;R_{G}C_{x}G_{L}\;V_{LINE}$$

(6) $$V_{RP}=V_{DC}-\omega_{R}\;R_{G}C_{x}G_{R}\;V_{R}-\omega_{R}\;R_{G}C_{in}G_{R}\;V_{R}$$

The microcontroller makes the difference between the *DC* voltage $V_{DC}$ and $V_{LP}$, obtaining

(7) $$V_{LDC}=V_{DC}-V_{LP}=\omega_{L}\;R_{G}C_{x}G_{L}\;V_{LINE}$$

Moreover, it makes the difference between the *DC* voltage $V_{DC}$ and $V_{RP}$, obtaining

(8) $$V_{RDCk}=V_{DC}-V_{RP}=\omega_{R}\;R_{G}C_{x}G_{R}\;V_{R}+K_{R}$$

The term $K_{R}$ in Equation (8) is equal to $\omega_{R}\;R_{G}C_{in}G_{R}V_{R}$ and it is a constant term that arises for the presence of the input capacitance $C_{in}$. A calibration procedure, performed at an early stage by disconnecting the probe to make null the capacitance $C_{x}$, allows us to determine the value of $K_{R}$. By subtracting this value from Equation (8), the microcontroller is able to determine

(9) $$V_{RDC}=\omega_{R}\;R_{G}C_{x}G_{R}\;V_{R}$$

Equations (7) and (9) show that the quantity $V_{LDC}$, calculated by the microcontroller, depends on $V_{LINE}$ and $C_{x}$, while $V_{RDC}$ depends only on $C_{x}$. Thus, by using Equations (7) and (9), the microcontroller can calculate the line voltage, that is

(10) $$V_{LINE\_CALC}=\frac{V_{LDC}}{V_{RDC}}\;\frac{G_{R}}{G_{L}}\;\frac{\omega_{R}}{\omega_{L}}\;V_{R}$$

where $V_{R}$ and $\omega_{R}$ are known quantities because they are locally generated. $G_{L}$ and $G_{R}$ are also known quantities. On the other hand, $\omega_{L}$ can be measured by the microcontroller. It is interesting to observe that both $V_{LDC}$ and $V_{RDC}$ in Equation (10) depend on the circuit parameters $R_{G}$ and $C_{x}$, but this dependence vanishes in the ratio.

In order to improve the accuracy of the measurement, an initial calibration procedure can be effectively aimed at determining the gain, G, and the offset, $V_{OS}$, errors affecting Equation (10). The resulting data can be used to correct Equation (10) during normal operation, that is

(11) $$V_{LINE\_EST}=G\;V_{LINE\_CALC}+V_{OS}$$

where $V_{LINE\_EST}$ is the estimated value of the amplitude of the fundamental component of the line voltage.

## 3. Circuit Design

The minimization of the supply power consumption of the proposed analog front-end is here achieved not only through the use of the very simple architecture discussed in the previous section, but also through a power-optimized circuit design. It is based on a low voltage supply at $V_{DD}=3.3\;V$ and on low-power rail-to-rail operational amplifiers, i.e., the Microchip MCP6242. In order to minimize the drawbacks of the above choices, which consist of low signal-to-noise ratios and in low performance of integrated circuits, an accurate circuit design has been developed.

Firstly, it should be noted that the peak-to-peak voltage of the signal $v_{OA}$ should be lower than $V_{DD}=3.3\;V$. This limits the amplitude of the reference signal $v_{R}$, whose peak-to-peak value is chosen to be equal to $V_{R}=2\;V$. Moreover, the best accuracy is achieved if the two quantities to be measured, i.e., the first two terms in the right-hand side of Equation (1), are comparable. Taking into account that $V_{LINE}$ is about 300 times $V_{R}$, the frequency of the reference signal is chosen equal to about 300 times $\omega_{L}$, i.e., $\omega_{R}=2\pi 15,000$ rad/s. The reference signal $v_{R}$, with the desired amplitude $V_{R}$ and frequency $\omega_{R}$, is generated by a Wien bridge oscillator [23,24]. However, the reference signal could also be generated by exploiting the microcontroller to produce a square wave, which can be filtered to obtain a sinusoidal signal.

**Figure 3:**
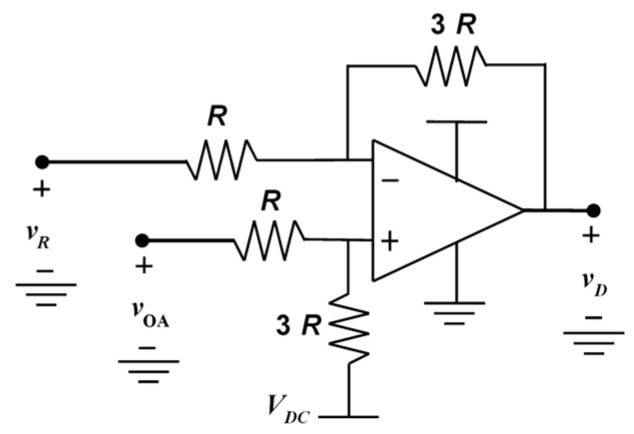
Schematic of the difference amplifier.

In order to keep the signal-to-noise ratio as large as possible, the gain resistor, $R_{G}$, of the input stage of the analog front-end is chosen equal to the largest value that does not saturate the operational amplifier, when the term $\omega_{L}C_{x}\;V_{LINE}$ assumes its maximum value. For the same reason, the gain of the difference amplifier, which is implemented through a bridge topology based on resistor arrays (Figure 3), is set equal to $G_{D}=3$. Moreover, to keep the signals as large as possible, the gains of the downstream filters, LPF and BPF, are chosen equal to $G_{F}=2$, leading to values for the overall gains equal to $G_{L}=G_{R}=6$.

**Figure 4:**
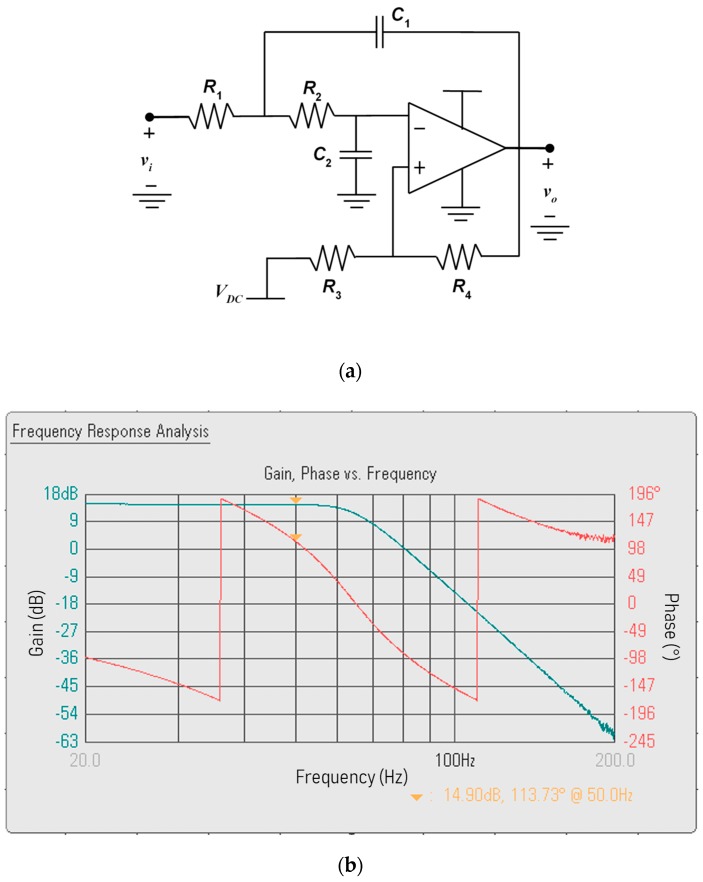
(**a**) Schematic of each one of the four stages of the line frequency 8th-order low-pass filter (LPF). (**b**) Measured Bode plot of the cascade of the difference amplifier and the 8th-order LPF.

At the output of the difference amplifier, the signal $v_{D}$ has a component at the line frequency, a component at the reference frequency, as shown in Equation (2), in addition to the line harmonics, which are amplified by the derivative behavior of the input stage. For this reason, the low-pass filter, LPF, should strongly attenuate all of the frequency components above the line frequency. An 8th-order Butterworth filter is designed with a cutoff frequency at 60 Hz and it is implemented through the cascade of the four Sallen-Key stages shown in Figure 4a. The values of the components of each stage are reported in Table 1 and the Bode plot of the overall transfer function, measured by a DSOX1204G-D1200BW1A Keysight Technology oscilloscope, is shown in Figure 4b.

**Figure 5:**
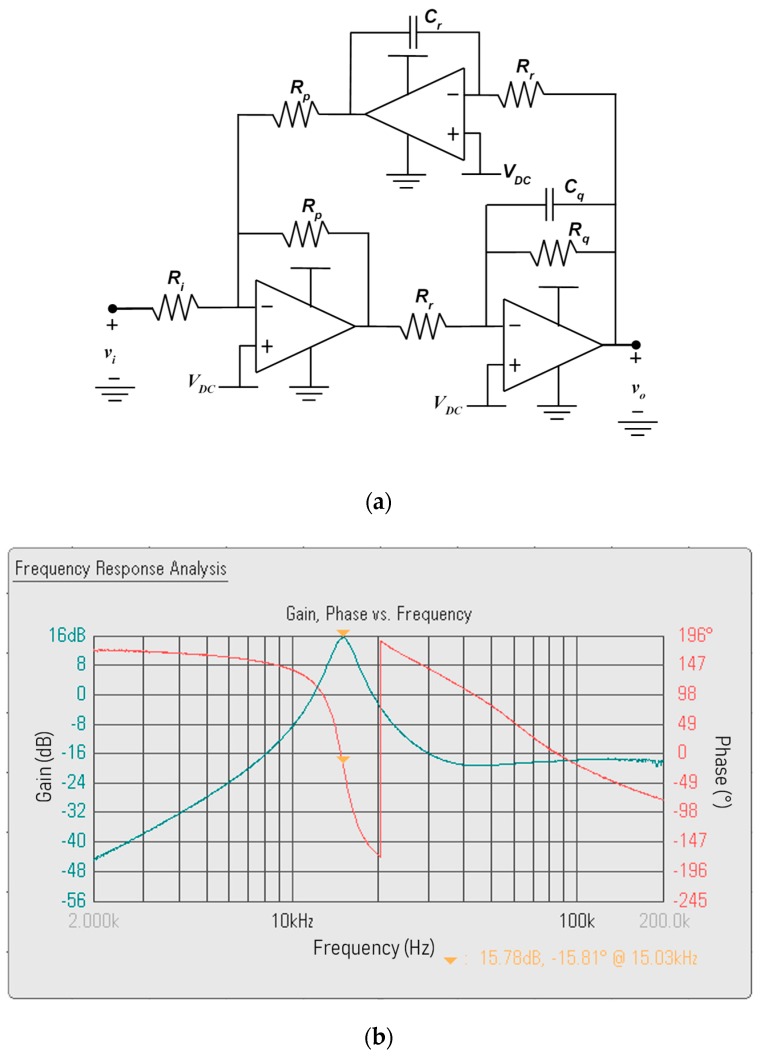
(**a**) Schematic of each one of the two stages of the reference frequency 4th-order band-pass filter (BPF). (**b**) Measured Bode plot of the cascade of the difference amplifier and the 4th-order BPF.

For extracting the component at the reference frequency from the signal $v_{D}$ at the output of the difference amplifier, a 4th-order band-pass filter is implemented through the two Tow-Thomas biquad stages in Figure 5a, in order to accurately control the bandwidth. This is a critical design aspect. As the line noise is amplified by the derivative behavior of the input stage, a very narrow-band is desired. However, a too narrow band leads to stability problems. The values of the components of each stage are reported in Table 2 and the Bode plot of the overall transfer function, measured by a DSOX1204G-D1200BW1A Keysight Technology oscilloscope, is shown in Figure 5b.

**Figure 6:**
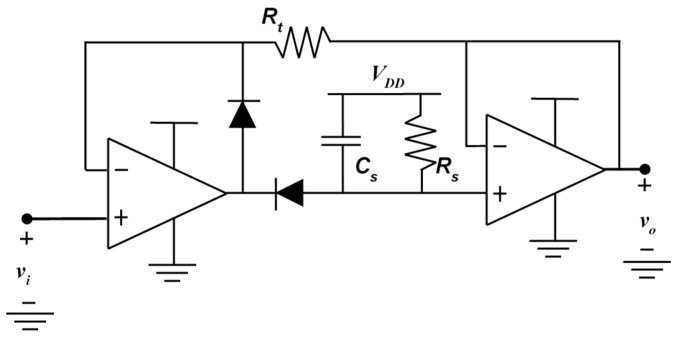
Schematic of the negative peak detectors.

With reference to the peak detector, the negative type is chosen, instead of the positive type, as shown in Figure 6, in order to obtain an output voltage that is lower than the DC voltage. Accordingly, a lower reference voltage can be chosen for the analog-to-digital converter (ADC), leading to a better quantization resolution. Moreover, in order to allow a fast discharge of the holding capacitor, the negative peak detectors have a reset input controlled by the microcontroller. As the capacitor is quickly charged by an operational amplifier and it is slowly discharged by a resistor, the reset control allows a quicker and accurate measurement.

## 4. Sensor Prototype

**Figure 7:**
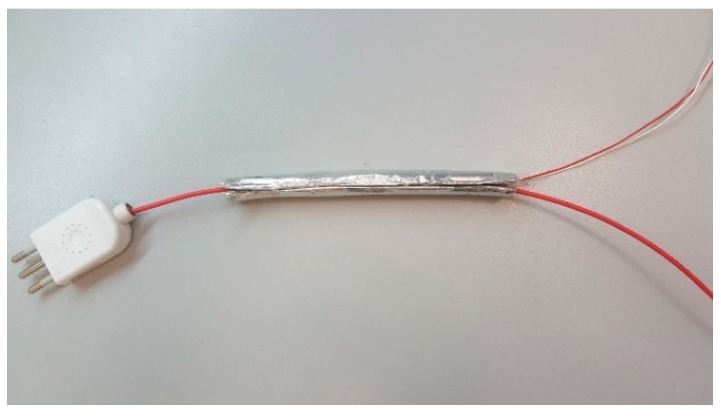
Prototype of the capacitive probe.

A prototype of the capacitive probe, shown in Figure 7, was made with a rubber tube of 1 cm diameter and 15 cm length, cut lengthwise to allow the insertion of the line cable. The tube was coated with silver paper both internally, to create the probe inner electrode, and externally, to create the probe outer shield.

**Figure 8:**
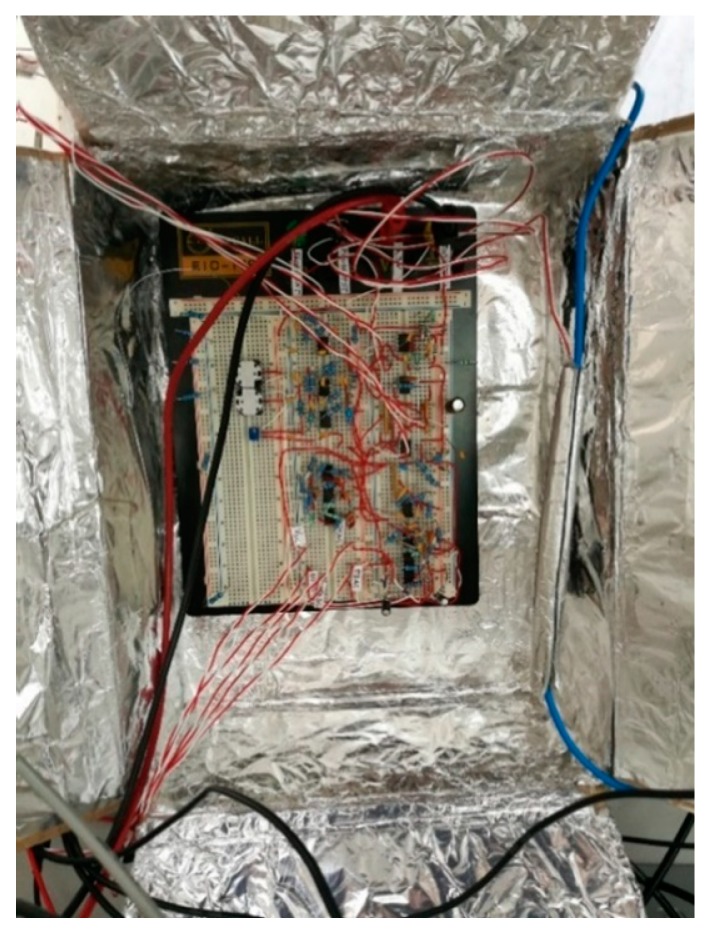
Prototype of the analog front-end with the capacitive probe inside a paper box that is internally coated with silver paper.

A prototype of the low-power analog front-end was implemented by means of a solderless breadboard, as shown in Figure 8. The value of the capacitance $C_{x}$, measured by the Keysight LCR (impedance) meter U1733C, is 14 pF, while the value of the capacitance $C_{S}$ is 140 pF. The whole prototype of the sensor was inserted in a paper box, internally coated with silver paper, in order to minimize the influence of the common mode voltage due to the mains.

**Figure 9:**
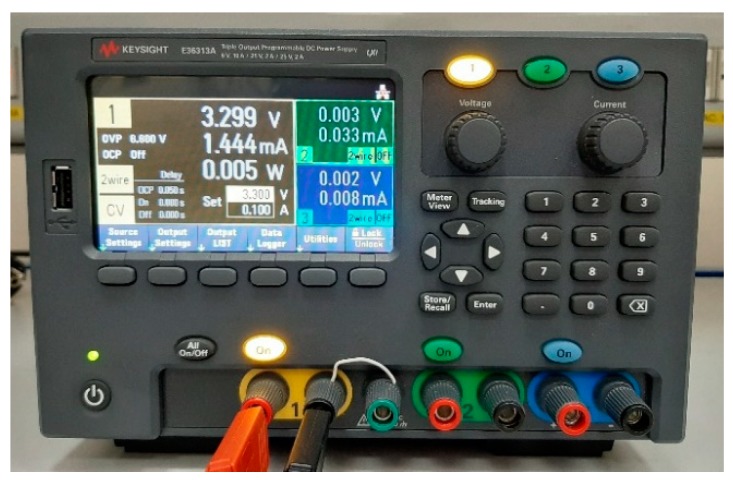
Power supply of the low-power analog front end, measuring the current drawn by the circuit.

The measured current drawn by the analog front-end supply is less than 1.5 mA, as measured by the Keysight E36313A shown in Figure 9. Thus, the power consumption is less than 5 mW, that is 100 times lower than the power consumption of other analog front-ends presented in literature for contactless power-line voltage measurement systems. In particular, the full analog solution proposed in Reference [19] is characterized by a very high supply voltage and by a quite complex circuit topology based on signal down-conversion by mixing. Indeed, the data reported in Reference [19] allows us to deduce that the analog multiplier forces the supply voltage to be greater than 16 V and the proposed circuit is composed by about 10 integrated circuits drawing a mean quiescent current of about 3 mA each one. Furthermore, the full digital solution proposed in Reference [22] is power-hungry, because it is based on a quite complex digital signal processing (filtering, integration, scaling, etc.) that requires both high-frequency and high-performing ADC converters as well as a real-time signal processing by an ARM Cortex-M4F microcontroller. On the other hand, the proposed solution is aimed at simplifying the analog processing and, at the same time, at minimizing the microcontroller operation, which only performs low-frequency and low-power analog to digital conversions.

## 5. Sensor Characterization

### 5.1. Measurement Setup

**Figure 10:**
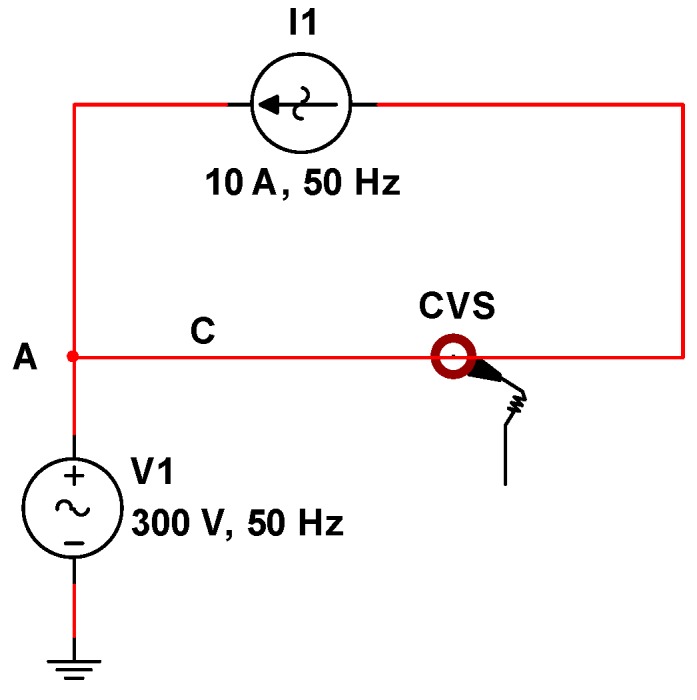
Virtual load connection for the characterization of the contactless voltage sensor (CVS).

The measurement setup used for the characterization of the sensor is able to generate *AC* voltages and currents up to, respectively, 300 V and 15 A root mean square (rms) values [25,26]. Voltage and current are separately generated and a virtual load connection scheme, shown in Figure 10, was used. In fact, the CVS should be sensitive only to electric fields; however, in actual situations, current flows into a power line, generating a magnetic field that could perturb the CVS operation.

In Figure 10, the CVS is inserted around the conductor C, which has a potential of 300 V with respect to the ground of the system, imposed by the generator V1. In the same conductor, a current of 10 A, imposed by the generator I1, flows. This constitutes a realistic condition to test the performance of the CVS.

From the point of view of the generation system, the configuration shown in Figure 10 forces the current generator I1 to work with a non-negligible common mode voltage, imposed by the voltage generator V1. In fact, considering the numerical example shown in Figure 10, one of the two terminals of I1, that is the node A, is at 300 V and the other cannot be connected to ground. This a requirement that, typically, commercial instrumentation does not satisfy.

**Figure 11:**
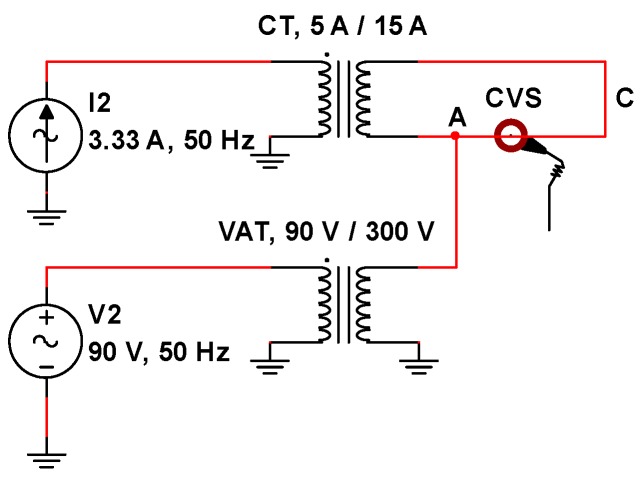
Virtual load connection, by means of a current transformer (CT) and a voltage autotransformer (VAT) for the characterization of the CVS.

Therefore, in order to overcome this issue, the voltage and current generator were provided of output transformers, realizing the scheme shown in Figure 11.

As for the voltage, an elevator autotransformer (VAT in Figure 11) with variable ratio was used—for the scope of this paper a ratio of about 3.33 was chosen. As for the current, a wound type current transformer (CT in Figure 11) for low voltage systems, with ratio of 15 A/5 A and rated burden of 20 VA, was used: The output of the current generator I2 was connected to the secondary winding, while the primary winding was short-circuited with the conductor C. The CVS was inserted around the conductor C and this was connected to the output of the VAT (i.e., the node A). In this way, the connection shown in Figure 11 is able to provide the same functionality of the scheme in Figure 10.

**Figure 12:**
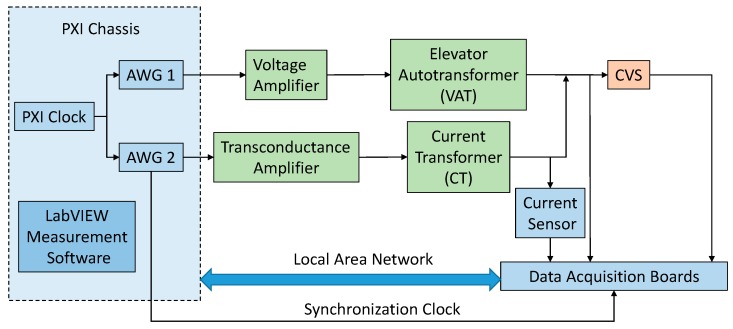
Block scheme of the measurement setup.

The complete block scheme of the measurement setup is shown in Figure 12. A National Instruments (NI) PCI eXtension for Instrumentation (PXI) platform was used. Two arbitrary waveform generators (AWG), the NI PXI 5422 (±12 V, 16 bit, 200 MHz), generate the low voltage signals, which are then amplified. These AWGs are synchronized by using the 10 MHz PXI clock as input reference clock for their phase locked loop circuitry: Then, a generation frequency of 6.4 MHz was used for the generated signals. The voltage amplifier is the NF HSA4052 (±150 V, 2 A, 500 kHz) and the transconductance amplifier is the Kepco BOP 20-20 (four quadrants, ±20 V, ±20 A, 10 kHz, voltage-controlled or current-controlled output). Their outputs have been connected, respectively, at the input of the VAT and at the input of the CT, as shown in Figure 11; moreover, the outputs of the VAT and of the CT are connected as in Figure 11. The CVS is connected at the output of the current transformer (CT), as shown in Figure 11.

The current at the output of the CT is sensed by a current sensor Pearson Electronics Current Monitor 411 (50 A/5 V, 1 Hz–20 MHz, 1%).

As for the Data AcQuisition boards (DAQ), a NI 9225 (±425 V, 24 bit, 50 kHz) and a NI 9239 (±10 V, 24 bit, 50 kHz) were used. They are inserted in a NI CompactDAQ chassis, which receive the synchronization clock of 6.4 MHz from one of the AWG: From this clock, the sampling clock for the two DAQs is derived and fixed to 12.5 kHz. In this way, generation and acquisition are perfectly synchronized.

The NI 9225 was used to acquire the output of the VAT, that is, voltage sensed by the CVS, whereas the NI 9239 was used to acquire three signals provided by the CVS, that are $V_{LP}$, $V_{RP}$, and $V_{DC}$ as in Equations (5) and (6), and the output voltage of the current sensor.

The estimation of the peak value of the input voltage of the CVS is then obtained by applying:

1. Equation (7) to obtain $V_{LDC}$ from $V_{LP}$ and $V_{DC}$;
2. Equations (8) and (9) to obtain $V_{RDC}$ ($K_{R}$ is preliminarily measured following the procedure described in Section 2);
3. Equation (10) to obtain $V_{LINE\_CALC}$ ($\omega_{L}$ is known and $\omega_{R}$, $G_{L},$ and $G_{R}$ are preliminarily measured).

### 5.2. Test Description

Different kinds of tests were performed to evaluate the performance of the CVS.

In the first group (hereafter identified with LIN), sine waves with frequency of 50 Hz and amplitudes from 20 to 300 V were generated. The aim of this test was to verify the voltage dependence of the CVS, i.e., the linearity of the sensor.

In the second group (hereafter identified with FREQ), sine waves with fixed amplitude of 230 V and frequency variable from 49.5 to 50.5 Hz were generated. The aim of this test was to verify the dependence of the CVS output on the input frequency.

In the third group (hereafter identified with FH1), waveforms composed by a fixed fundamental tone, 230 V and 50 Hz, and a harmonic tone with frequency from 2nd to 3rd harmonic order and phase variable from −π rad to π rad were used. As for the amplitude, a fixed amplitude equal to the limits stated in the standard EN 50160 [27] (2% for 2nd harmonic and 5% for 3rd harmonic) was used. The aim of this test was to verify the influence of the lowest harmonic components on the measurement of the peak value of the fundamental component. In fact, a change of the harmonic angle reflects in a significant variation of the peak of the waveform and so, if the CVS low pass filter does not sufficiently attenuate the amplitude of the harmonic tone, then this causes a less accurate measurement. The reason why the analysis is limited to the 3rd harmonic is that the low-pass filter of the CVS has an attenuation of about 75 dB at 200 Hz (i.e., the frequency of the 4th harmonic); therefore, a 4th harmonic eventually present on the waveform is expected to have a negligible influence on the CVS performance.

In the fourth group (hereafter identified with FHN), waveforms composed by a fixed fundamental tone, 230 V and 50 Hz, and all the harmonic components, from the 2nd to the 50th order, were used. The amplitudes and the phase angles of the harmonics were randomly chosen; in particular, the phase is uniformly distributed between −π rad to π rad, whereas the amplitude is uniformly distributed between zero and the maximum value for that harmonic stated in [27]. Moreover, the maximum Total Harmonic Distortion (THD), according to Reference [27], was fixed to 8%. Since the waveforms of this group of tests represent realistic waveforms of a low voltage power system, testing the performance of the CVS in these conditions is highly important.

It is worthwhile noting that all the described tests have been performed with and without a current flowing in the power line. In particular, in the LIN and FREQ tests, a sinusoidal current with amplitude of 10 A (and the same frequency and phase angle of the voltage) has been used. In the FH1 and FHN tests, the current had a fundamental tone with amplitude of 10 A, frequency of 50 Hz and zero phase, whereas the spectral components had the same characteristics of the voltage components (i.e., the same orders, the same percentage amplitudes and the same phases).

### 5.3. Experimental Results

In all the tests executed on the CVS, for each generated waveform, ten time frames of 1 s (that is ten repetitions for each measurement) were acquired. Then, since generation and acquisition are synchronized, the reference quantity, i.e., the peak value of the fundamental component, was evaluated by mean of a discrete Fourier transform (DFT), using a rectangular window (that is with no window, since no spectral leakage is present). Thus, the ratio error of the CVS was evaluated as in Equation (12):

(12) $$\Delta R=\left(\frac{V_{LINE\_CALC}-V_{LINE\_REF}}{V_{LINE\_REF}}\right)\times 100$$

where $V_{LINE\_CALC}$ is the calculated input voltage obtained with the measured CVS output signals and $V_{LINE\_REF}$ is the reference value of the input obtained by means of the DFT.

As regards the LIN tests, ten amplitudes, from 20 V to 295 V were used.

According to what we stated at the end of Section 2, in order to improve the performance of the CVS, the LIN tests were also used for the determination of the gain, G, and the offset, $V_{OS}$, errors affecting Equation (10). They have been found by minimizing the fitting root mean square error of Equation (11) to experimental data. In this way, $V_{LINE\_EST}$ is obtained from $V_{LINE\_CALC}$; also, Equation (12) is then modified by introducing $V_{LINE\_EST}$.

(13) $$\Delta R=\left(\frac{V_{LINE\_EST}-V_{LINE\_REF}}{V_{LINE\_REF}}\right)\times 100$$

It is worthwhile noting that G and $V_{OS}$ are used in all the following presented results, to correct the measured data.

**Figure 13:**
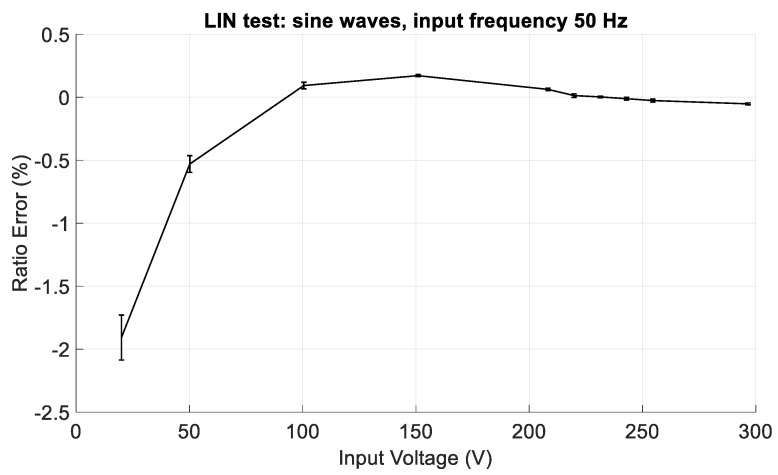
Ratio error in the LIN tests, with sinusoidal input voltage with frequency of 50 Hz and amplitudes from 20 V to 295 V

**Figure 14:**
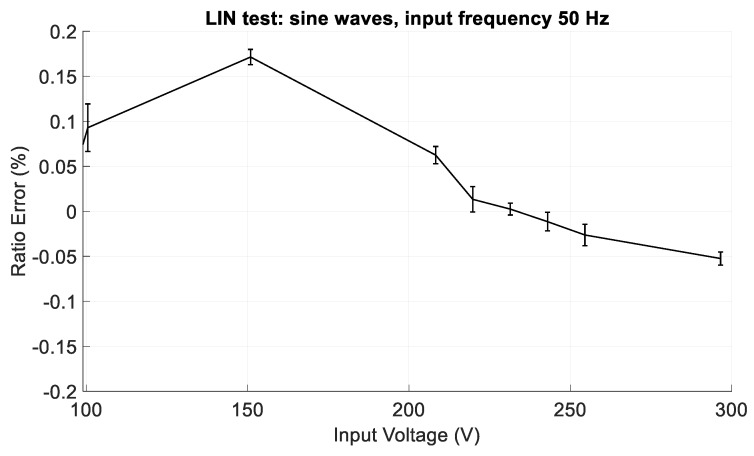
Zoom of Figure 13: ratio error in the LIN tests, with sinusoidal input voltage with frequency of 50 Hz and amplitudes from 100 V to 295 V

Figure 13 shows the ratio error in the LIN tests, from 20 V to 295 V, whereas a zoom is shown in Figure 14, where the ratio error in the LIN tests, from 100 V to 295 V is depicted. The error bars represent the expanded measurement uncertainties (level of confidence equal to 95%). The CVS, in general, behaves as a non-linear device, with a ratio error which exceeds 2% at low input values. However, from about the 30% of the input range up to the full-scale value, it has good performance, since the ratio error is lower than ±0.2%, including the expanded uncertainty.

As for the current influence, the results are not reported here for sake of brevity: in fact, the measured variations in presence of current were much lower than the measurement uncertainty. For a rigorous evaluation of the measurement uncertainty, the variations due to the presence of current have been considered a repeatability contribution and included in the uncertainty evaluation [28]. This consideration is valid for all the other performed tests; therefore, this will not be repeated in the following.

**Figure 15:**
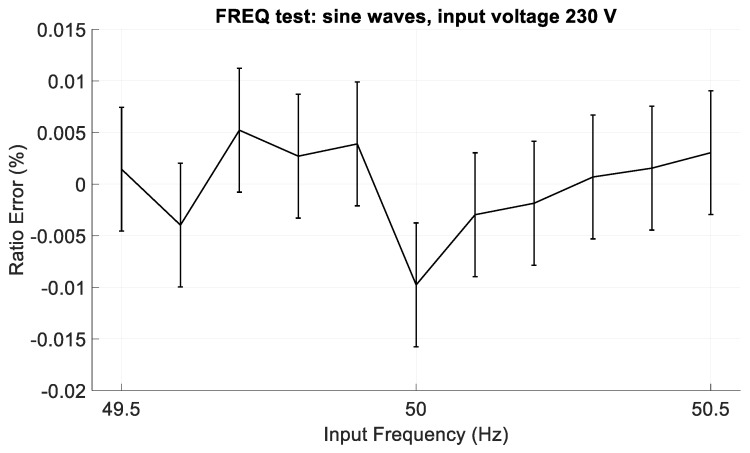
Ratio error in the FREQ tests, with sinusoidal input voltage with amplitude of 230 V and frequencies from 49.5 Hz to 50.5 Hz.

As regards the FREQ tests, 11 frequency values, from 49.5 to 50.5 Hz were chosen. Figure 15 shows the ratio error in the FREQ tests. In this case, the CVS exhibits very good performance, since the ratio error is lower than ±0.02%, considering also the expanded uncertainty.

**Figure 16:**
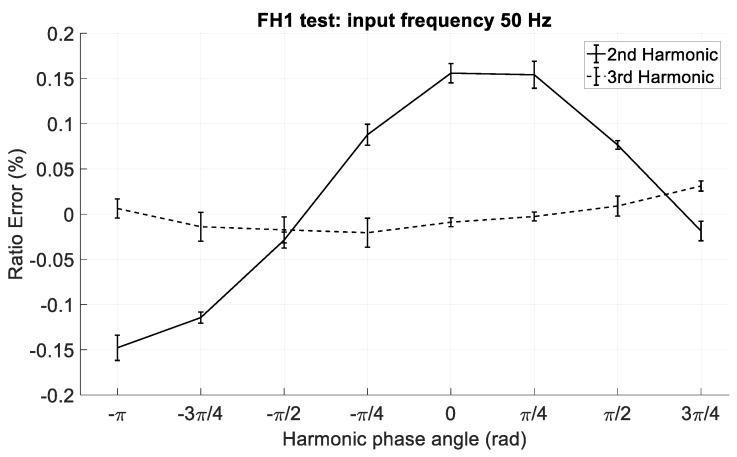
Ratio error in the FH1 tests; waveforms have two tones, fundamental tone has amplitude of 230 V and frequency of 50 Hz, the harmonic tone amplitude of 2% (5%), harmonic order 2 (3) and variable phase.

As regards the FH1 tests, eight phase values, from -π rad to 3π/4 rad were chosen. Figure 16 shows the ratio error in the FH1 tests. As is already highlighted in Section 5.2, the harmonic phase determines significant variations of the waveform peak value. In fact, especially for the 2nd harmonic, the variation of the phase angle determines a variation of the measured peak value of the fundamental component, which, instead, remains constant in all the FH1 tests. This is due to the fact that the low pass filter attenuates the 2nd harmonic of about 30 dB (a factor of about 32) whereas attenuates the 3rd harmonic of about 55 dB (a factor of about 560). Summing up, we can state that the maximum ratio error in presence of a 2nd harmonic is lower than ±0.2%, whereas in presence of a 3rd harmonic it is lower than ±0.05%.

**Figure 17:**
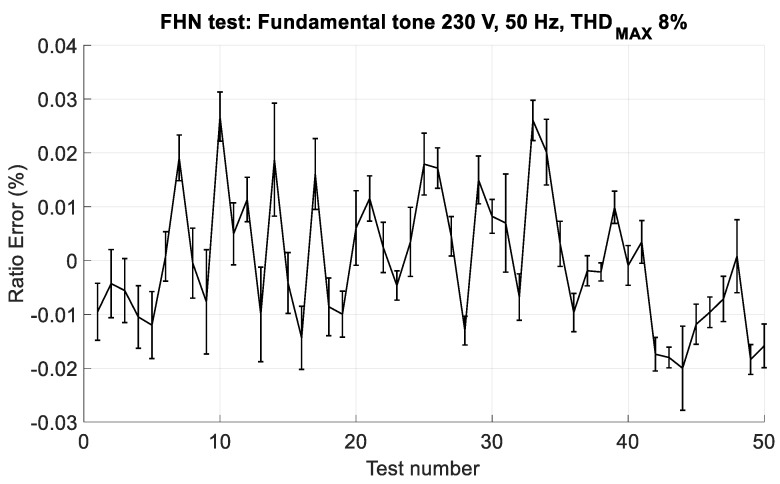
Ratio error in the FHN tests; waveforms are composed of a fundamental tone, with amplitude of 230 V and frequency of 50 Hz, and all the harmonic tones, from the 2nd up to the 50th, with random amplitude and phase. The maximum total harmonic distortion (THD) is 8%.

As regards the FHN tests, 50 different waveforms were generated, with random amplitudes and phases for the harmonic tones and a maximum THD of 8%. Figure 17 shows the ratio error in the FHN tests. The maximum ratio error is lower than ±0.04%, confirming the results of the FH1 tests, i.e., the harmonic components with order higher than the 3rd have a negligible influence on the CVS performance.

Summarizing, combining the results of all the tests, we can state that, for a generic power system voltage waveform, that is with amplitude between 100 and 300 V, frequency between 49.5 and 50.5 Hz and random harmonic distortion (THD lower than 8% according to Reference [27]) the ratio error is lower than ±0.3%.

## 6. Conclusions

This paper presents a new low power contactless voltage sensor for low voltage power systems. It is made of a capacitive probe, which surrounds the power cable, and a low power analog conditioning circuit. The minimization of the supply power consumption of the proposed analog front-end is here achieved not only through the use of a very simple architecture, but also through a power-optimized circuit design. It is based on a low voltage supply at 3.3 V and on low-power rail-to-rail operational amplifiers. The current drawn by the analog front-end supply is less than 1.5 mA and, thus, the power consumption is less than 5 mW, that is 100 times lower than the power consumption of other analog front-ends presented in literature for contactless power-line voltage measurement systems.

The sensor has been characterized with a specifically realized measurement setup, able to verify also the influence of a current flowing into the power cable on the measurement accuracy, and it was tested in the most common operating conditions of a low voltage power system. The maximum ratio error of the sensor is ±0.3% from 100 to 300 V.

Other than the possibility to measure voltage without contact, it has the feature of low power consumption, which makes it attractive for IoT applications.

## Figures and Tables

**Table 1:** Components of the line-frequency low-pass filter in Figure 4.

	First Stage	Second Stage	Third Stage	Fourth Stage
*R_1_* (kΩ)	33.2	17.8	13.0	4.32
*R_2_* (kΩ)	37.4	26.1	16.5	6.04
*R_3_* (kΩ)	69.8	∞	∞	∞
*R_4_* (kΩ)	69.8	0	0	0
*C_1_* (nF)	56.0	150	330	2700
*C_2_* (nF)	100	100	100	100

**Table 2:** Components of the reference-frequency band-pass filter in Figure 5.

	First Stage	Second Stage
*R_i_* (kΩ)	21	21
*R_p_* (kΩ)	10	10
*R_r_* (kΩ)	10	10
*R_q_* (kΩ)	30	30
*C_q_* (nF)	1.056	1.056
*C_r_* (nF)	1.056	1.056
